# SurA-like and Skp-like Proteins as Important Virulence Determinants of the Gram Negative Bacterial Pathogens

**DOI:** 10.3390/ijms24010295

**Published:** 2022-12-24

**Authors:** Donata Figaj, Patrycja Ambroziak, Iga Rzepka, Joanna Skórko-Glonek

**Affiliations:** Department of General and Medical Biochemistry, Faculty of Biology, University of Gdansk, Wita Stwosza 59, 80-308 Gdansk, Poland

**Keywords:** SurA, Skp, periplasmic folding factors, virulence factors, protein folding, PQCS, secretion systems, protein export, Gram-negative pathogens

## Abstract

In the Gram-negative bacteria, many important virulence factors reach their destination via two-step export systems, and they must traverse the periplasmic space before reaching the outer membrane. Since these proteins must be maintained in a structure competent for transport into or across the membrane, they frequently require the assistance of chaperones. Based on the results obtained for the model bacterium *Escherichia coli* and related species, it is assumed that in the biogenesis of the outer membrane proteins and the periplasmic transit of secretory proteins, the SurA peptidyl–prolyl isomerase/chaperone plays a leading role, while the Skp chaperone is rather of secondary importance. However, detailed studies carried out on several other Gram-negative pathogens indicate that the importance of individual chaperones in the folding and transport processes depends on the properties of client proteins and is species-specific. Taking into account the importance of SurA functions in bacterial virulence and severity of phenotypes due to *surA* mutations, this folding factor is considered as a putative therapeutic target to combat microbial infections. In this review, we present recent findings regarding SurA and Skp proteins: their mechanisms of action, involvement in processes related to virulence, and perspectives to use them as therapeutic targets.

## 1. Introduction

The success of a pathogenic bacterium to infect the host and develop disease symptoms depends on the efficient and coordinated action of an appropriate repertoire of virulence factors. The following features should be mentioned: (1) motility and chemotaxis (finding a suitable niche); (2) ability to adhere (binding to surfaces on/in the host’s body); (3) ability to transmigrate through the epithelial layers; (4) evasion of the host immune systems (passive and active, including modulation of host immune systems); (5) production of toxins. For details, please refer to the excellent reviews [1,2,3,4,5,6,7].

The vast majority of virulence factors are proteins exposed on the bacterial cell surface or secreted extracellularly. Cells of the Gram-negative bacteria are surrounded by two membranes, the outer (OM) and inner (cytoplasmic; IM) membranes, which encompass a periplasmic space with a peptidoglycan layer [8]. The consequence of such a cell structure is complexity of protein export. In the case of many virulence factors, bacteria use dedicated transport systems that secrete their substrates directly outside the cell, bypassing the periplasm. However, many important virulence determinants are first transported to the periplasm, where they can mature and eventually pre-fold, followed by their migration to the OM. There, they either are incorporated into the membrane structures (e.g., adhesins, nutrient uptake systems, flagella elements) or are secreted outside the cell. Alternatively, virulence factors may become a part of the outer membrane vesicles (OMVs) and thus leave the bacterial cell [9].

In many cases, secretory proteins that require periplasmic transit should be assisted by components of the so-called Protein Quality Control System (PQCS), which include periplasmic chaperones, folding catalysts and proteases. PQCS protects client proteins against improper interactions and allows them to obtain correct structure; alternatively, it maintains a protein in a structure suitable for incorporation into membranes or further translocation through the OM. Moreover, PQCS prevents the formation of protein aggregates and removes misfolded proteins [10,11,12]. These functions become particularly important under stressful conditions which are typically encountered by a pathogen during infection. The host’s organism, in response to the appearance of pathogenic organisms, activates defense mechanisms leading inter alia to the appearance of many stressors (e.g., increased temperature, reactive oxygen and nitrogen species) [13]. Under stressful conditions, the achievement and maintenance of the native protein structure are particularly challenging. Therefore, disturbances in the functioning of PQCS often lead to a reduction in the virulence (or its complete lack) of pathogenic strains [14,15].

This review presents current data on the homologs of two crucial periplasmic protein folding factors, SurA, a protein with dual chaperone and peptidyl–prolyl isomerase (PPIase) activity, and the chaperone Skp. A particular emphasis is put on folding and export of bacterial virulence factors. The recent discoveries involving consequences of SurA or Skp deficiency on bacterial virulence are provided, as well as perspectives to use these proteins as targets for development of new antimicrobial strategies.

## 2. Export of Virulence Factors

**Figure 1:**
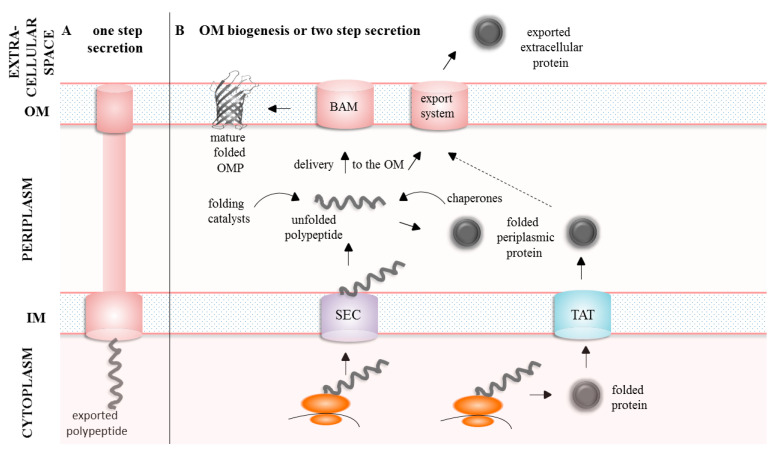
Overview of mechanisms related to the export of proteins. (**A**). In the one-step secretion systems, the secretory proteins are transported directly outside the cell. (**B**). In the two-step secretion systems, newly synthesized proteins are transported through the IM to the periplasm by the SEC (unfolded proteins) or TAT (folded proteins) machineries. Once they reach the periplasm, unfolded proteins acquire their final conformation with the participation of the folding catalysts. Alternatively, proteins destined to the OM or extracellular environment remain unfolded and are kept in a conformation suitable to enter/cross the OM by the periplasmic chaperones. The latter proteins are then exported outside the cell by sophisticated secretion systems or become incorporated into the OM by the BAM machinery.

Bacterial proteinaceous virulence factors can reach their final destination in several ways using one of the following export strategies (summarized in the Figure 1):

1. Direct (one-step)—proteins can be secreted from the cytoplasm directly outside the cell (bypassing the periplasm) using dedicated transport systems (e.g., type 1, 3 or 4 secretions systems) (reviewed in [16,17]).
2. Two-step—proteins first pass through the inner membrane (using the SEC or TAT system), traverse the periplasm, reach the outer membrane (OM), where they are either incorporated into the OM with the participation of the β-barrel assembly machinery (the BAM complex) or exported outside the cell by dedicated secretory systems (e.g., type 2, 5, 8 or 9 secretion systems). Both SEC and TAT substrates contain cleavable N-terminal signal sequences which are cleaved during or shortly after translocation. However, the TAT system translocates folded substrates, whereas the SEC substrates are translocated in unfolded state [18,19].

The two-step export is used to translocate many virulence factors. These include (1) the integral outer membrane proteins (OMPs) such as adhesins, transporters (nutrient acquisition in limited environments) and antibiotic efflux pumps and (2) extracellular factors such as large filamentous structures on the surface of a bacterial cell (pili, fimbriae, and curli), nonfimbrial adhesins, toxins, iron/heme-acquisition systems, cytolysins/haemolysins and proteases (reviewed in [20,21]).

The two-step export is associated with a number of challenges. To name the most important ones, (1) proteins leave the SEC channel in an unfolded form [19]. Therefore, they are prone to abnormal non-specific interactions and eventually may aggregate. (2) Proteins destined for the outer membrane (OMPs) or those that should be secreted outside the cell, in many cases do not obtain their native conformation during periplasmic transport and the functional structure is achieved at their final destination [22,23,24]. Moreover, the OMPs fold into beta barrel structures composed of 8–36 beta strands [25], but they can additionally contain separately folded extracellular or periplasmic domains [26]. Therefore, folding of OMPs is a particularly complex process in which the intra-membrane and extra-membrane domains acquire their final structure usually independently.

## 3. Two-Step Bacterial Secretion Systems

### 3.1. The Type Five Secretion System (T5SS)

**Figure 2:**
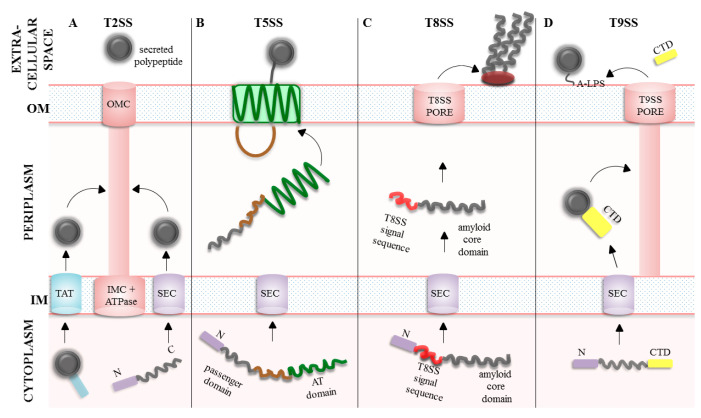
Two-step secretion systems in Gram-negative bacteria. The secretory preproteins (marked in dark grey) are directed to the SEC or TAT translocation systems located in the IM. The N-terminal signal sequences (marked in purple or blue) direct the preproteins to the appropriate transport systems (SEC or TAT, respectively) and are subsequently cleaved off. Once in the periplasm, the secretory proteins exit the cell by one of the following secretion systems: (**A**) In the T2SS, the cargo proteins become folded in the periplasm and then they are transported via pseudopilus, OMC (outer membrane complex) and IMC (inner membrane complex) structures across the OM. (**B**) In the T5SS, the “translocator” domain (green) is inserted into the OM and the “passenger” domain (grey) is translocated across the OM where it acquires its final conformation. Both domains are separated by a linker sequence (brown). (**C**). In the T8SS, the substrate curli subunits are maintained in an unfolded state in the periplasm. The T8SS signal sequence (red) directs the amyloid core domain (grey) to the T8SS channel, the substrate is secreted and remains anchored to the OM to form amyloid fibers. (**D**) In the T9SS, the secretory protein acquires its final conformation in the periplasm and is directed to the T9SS channel by the long C-terminal domain (CTD) (yellow). After translocation, the CTD is cleaved off and the substrate protein remains anchored to the OM via A-LPS.

The T5SS is one of the most common secretion systems among Gram-negative bacteria and the proteins of the T5SS are often called autotransporters (AT). The proteins secreted by the T5SS are diverse in size, structures and function (adhesins, toxins, enzymes) [27,28]. Generally, a protein to be exported termed a “passenger” requires assistance of the dedicated OM β-barrel protein, the “translocator”. In the simplest case, the T5SS consists of only one polypeptide chain with a β-barrel translocation domain in the membrane and an extracellular passenger or effector region. The precursors of AT pass through the IM via the SEC machinery and travel across the periplasm to reach the OM. At the OM, the “translocator” becomes inserted and folded in the lipid bilayer by the BAM and/or translocation and assembly module (TAM) machineries, and subsequently the “passenger” domain is translocated to the cell surface. There, the “passenger” adopts its functional conformation. Some “passenger” proteins are released into extracellular milieu while other remain covalently attached to the bacterial surface (reviewed in [27,29,30]). Depending on the exact domain architecture of the protein, the T5SS can be further divided into subgroups referred to as type Va to f (reviewed in [27,30]). Briefly, the Va type is recognized as a classical autotransporter and is made up of a single polypeptide containing two major domains: the N-terminal “passenger” domain and the C- terminal “translocator” β-barrel. Vb, also called the two-partner secretion system (TPSS), consists of two separate polypeptide chains encoded in one operon: “translocator” (TpsB) with two periplasmic polypeptide transport-associated (POTRA) domains and “passenger” (TpsA). Vc is very similar to the Va type, however it forms trimeric structures and is therefore referred to as trimeric autotransporter adhesins (TAAs). Vd possesses features of both Va and Vb systems. Vd and Vb “translocator” domains are similar, however in Vd there is only one POTRA domain, whereas Vb type has two POTRA domains. The difference between the “passenger” domains of Vd and Va type is that the Vd “passengers” characterized so far function mainly as lipases/esterases and the “passengers” in the Va type have a wide range of functions. Ve functions similarly to the Va type, however its domain organization is inverted (the N-terminal “translocator” domain and the C—terminal “passenger” domain). Moreover, the “translocator” domain in the Ve type may contain an extra periplasmic domain. Recently, another secretion mechanism, unique for bacterium *Helicobacter pylori*, has been proposed as a Vf type. Its “passenger” domain is a surface-exposed domain which is inserted between the N-terminal strands of the “translocator” β-barrel. In fact, the “passenger” is a large, extracellular loop of a β-barrel which is folded into a domain [31]. This topological feature is very atypical for the T5SS; therefore, the affiliation of Vf to autotransporters is questionable (Figure 2) [30].

Most of the known T5SS substrates are virulence factors (reviewed in [30,32]), including *Neisseria gonorrhoeae* immunoglobulin A protease (responsible for degradation of host antibodies), *Shigella flexneri* IscA autotransporter (responsible for intracellular motility and adhesion to host cells) or VacA protein (one of the major virulence factors in *H. pylori*) [30,33,34,35].

### 3.2. The Type 2 Secretion System (T2SS)

The T2SS consists of an OM complex called secretin, periplasmic pseudopilus, an IM complex and cytoplasmic ATPase. This sophisticated system contains 12–15 proteins that are often encoded in a single operon [36,37]. Briefly, substrates with signal sequences are transported into the periplasm by the SEC or the TAT systems [17]. Once in the periplasm, the protein is folded and then exported out of the cell by the T2SS. Despite many studies, the mechanism by which the secretory proteins leave the cell is poorly understood. The substrate proteins do not possess any characteristic signal sequences directing them to the T2SS, thus recognition mechanism seems to be individual for each substrate and is most probably based on structural motifs [9,38]. According to one of the models [39], the already folded substrate interacts with periplasmic domain of secretin. This interaction leads to activation of ATPase and retraction of pseudopilus which then pushes substrates through the secretin channel (Figure 2). The T2SS is an important virulence factor, and many pathogens utilize this pathway to deliver toxins to target cells. For example, *Vibrio cholerae* uses T2SS to export the cholera toxin during infection. In *P. aeruginosa*, the T2SS transports several virulence determinants, e.g., exotoxin A which blocks host cells protein synthesis [40,41,42].

### 3.3. The Type 8 Secretion System (T8SS)

The T8SS refers to the curli biogenesis pathway and is based on the nucleation–precipitation mechanism. The curli subunits are secreted as largely unstructured proteins that undergo transition to β-rich structures forming regular fibers only at the cell surface. Curli subunits have the N-terminal signal sequences and are transported through inner membrane by the SEC machinery. As subunits reach the periplasm, they are maintained in an unfolded state by periplasmic chaperones which facilitate secretion by the T8SS outer membrane channel. After secretion, curli fibers remain anchored to the OM where they assemble into curli polymers (Figure 2) [28,43]. The curli fibers form scaffolds that provide adhesive and structural support necessary in multicellular community in biofilms. In some pathogenic bacterial species, curli are implicated in host colonization, cell invasion and activation of innate responses (reviewed in [43]).

### 3.4. The Type 9 Secretion System (T9SS)

The recently discovered T9SS, also known as a Por secretion system (PorSS), is specific to the Bacteroidetes phylum. The flagship representative of Bacteroidetes, *Porphyromonas gingivalis* (the bacterium associated with periodontitis), utilizes the T9SS to export many proteins, including its gingipain virulence factors, which cause damage to the host tissues and modulate the host’s immune response. Additionally, this system is also required for gliding motility of *Flavobacterium johnsoniae* (reviewed in [44,45,46]).

The secretory proteins of the T9SS have the N-terminal signal peptides directing to the SEC system and long conserved C- terminal domain signals (CTD) which target them to the OM translocon. During translocation, the N-terminal signal sequence is cleaved, and the substrate is released to the periplasm where it acquires its stable conformation. After passing through the OM, the CTD is cleaved, and the functional secretory protein is anchored covalently by A-LPS (an anionic polysaccharide, one of the two LPS forms identified in *P. gingivalis*) to the OM (Figure 2) [44].

## 4. Importance of the Periplasmic Folding Factors for the Efficient Transport and Secretion of Proteins

As mentioned in the Introduction, the components of the PQCS maximize the efficiency of transport/export of proteins mainly by preventing unfavorable interactions of unfolded proteins and maintaining their structure competent for further transport or incorporation into the OM. In the model Gram-negative bacterium, *E. coli*, it is assumed that the peptidyl–prolyl isomerase/chaperone SurA plays a major role in the trafficking and incorporation of the OMP proteins into the OM and it directly interacts with the BAM complex [47,48,49]. The second alternative delivery pathway involves chaperone Skp and possibly DegP/HtrA protease/chaperone. In the case of proteins exported by the two-step types of secretion systems, it is also presumed that some cargo proteins require the SurA or/and Skp chaperones to assist their folding and guide them to the OM translocon.

### 4.1. The SurA Protein

**Figure 3:**
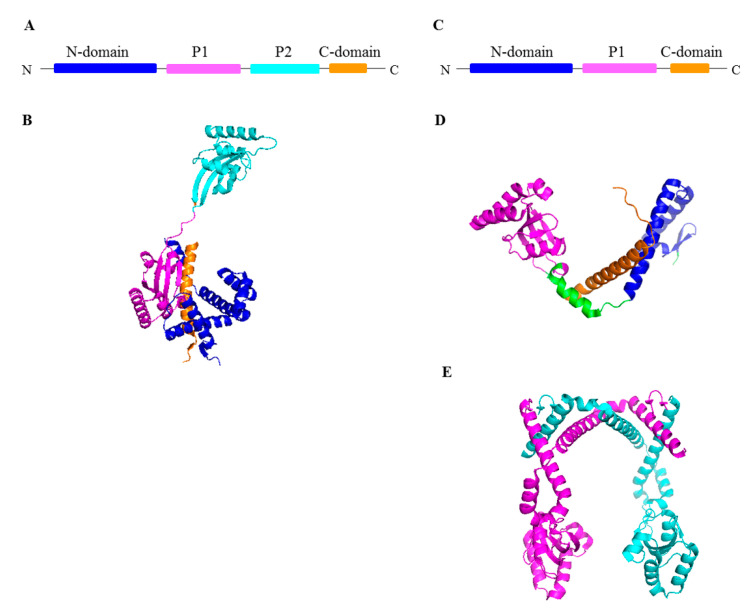
Structures of SurA from *E. coli* (**A**,**B**) and a SurA-like protein, HP-0175 from *H. pylori* (**C**–**E**). (**A**,**C**) Linear diagrams of SurA proteins. (**B**,**D**) Ribbon representation of the X-ray structures of monomers. Each domain is marked with a different color, corresponding to the color shown in the linear sequence. (**E**) Hp_0175 dimer, each monomer is shown in a different color. The image was prepared using the PDB files: 1 m5y (**B**), 6 bhf (**D**) and 5 ez1 (**E**).

SurA belongs to the parvulin class of PPIases. It was first identified as essential for survival in the stationary phase of *E. coli*; hence, it was named “Survival protein A” [50,51]. *E. coli* SurA is induced under stressful conditions by the pathway dependent on the alternative RNA polymerase subunit, sigma E [52]. Subsequent studies demonstrated importance of SurA in processes related to OMP trafficking and insertion into the OM. SurA exhibits two independent activities, PPIase and chaperone [53,54,55], which are located at distinct domains/modules of the protein. The *E. coli* SurA protein is composed of four domains connected by flexible linkers: the N-terminal domain (N-domain), two parvulin-like PPIase domains (P1, P2) and the C-terminal domain (C-domain), of which the N-terminal and C-terminal domains form a core chaperone module (Figure 3A,B) [56,57]. The orientations of the P1 and P2 domains relative to the core domain are dynamic and are proposed to oscillate between the “open” states and conformations in which the core domain and P1 or P2 are in close proximity [47,58,59]. The SurA homologs from other bacteria (frequently termed the SurA-like proteins) are also composed of the core chaperone modules (comprising the N- and C-terminal domains) and one or two PPIase domains. The *H. pylori* HP_0175 is an example of the SurA-like proteins with only one PPIase domain (Figure 3C–E). Initially, it seemed that the P1 and P2 domains are dispensable for the chaperone activity because a variant of SurA lacking both PPIase domains retains chaperone function in vivo [48]. However, recent findings point to importance of the P1 and P2 domains in processes related to the delivery of OMPs to the BAM machinery and subsequent incorporation of β-barrels into the OM. First, SurA lacking both or one PPIase domains is not able to prevent aggregation of the OmpT protein, demonstrating that these domains are important to chaperone at least some client proteins [60]. Second, the P2 domain participates in binding of SurA to the periplasmic regions of BAM and modulates OMP folding [47].

Nonetheless, the PPIase activity does not seem to be essential for SurA function in OMP assembly [48,61] and SurA functions predominantly as a chaperone in the OMP biogenesis. In *E. coli*, SurA is a primary chaperone for many β-barrel OMPs (e.g., LamB, OmpA, OmpC, OmpF), and its deletion results in their reduced cellular content, leading to disturbances in the OM [49,53,55]. Moreover, folding of the LptD protein was shown to be strictly dependent on SurA [62]. LptD is an essential OM β-barrel protein that is required for the lipopolysaccharide (LPS) insertion into the OM [63]. Therefore, a lack of SurA leads to impairment of LptD biogenesis, and this way it indirectly induces changes in LPS assembly and insertion [62].

SurA forms contacts with its OMP clients at multiple sites localized mainly on the N-terminal part of the core domain, but also on the P1 domain [58] and shows specificity toward Ar-X-Ar sequences in a substrate (where Ar is an aromatic residue and X is any amino acid residue) [64,65,66,67]. The Ar-X-Ar sequences are abundant in OMPs particularly within the C-terminal beta strands. Therefore, it was postulated that SurA binds mainly the C-terminal parts of the OMP clients (reviewed in [68]). Indeed, a peptide mimicking the typical C-terminus of OMPs was bound with a high affinity by SurA [67]. However, precise chemical cross-linking–mass spectrometry experiments demonstrated that the interactions between SurA and its clients occur in regions rich in tyrosine residues that are found at different sites on the substrates, and that their exact locations are specific to a particular protein. For example, unfolded OmpA is bound mainly at the N-terminal central part, while the C-terminus does not seem to form contacts with SurA [58]. On the other hand, OmpX is bound mainly via its central part, but SurA-OmpX C-terminus interactions were also detected [58,69].

**Figure 4:**
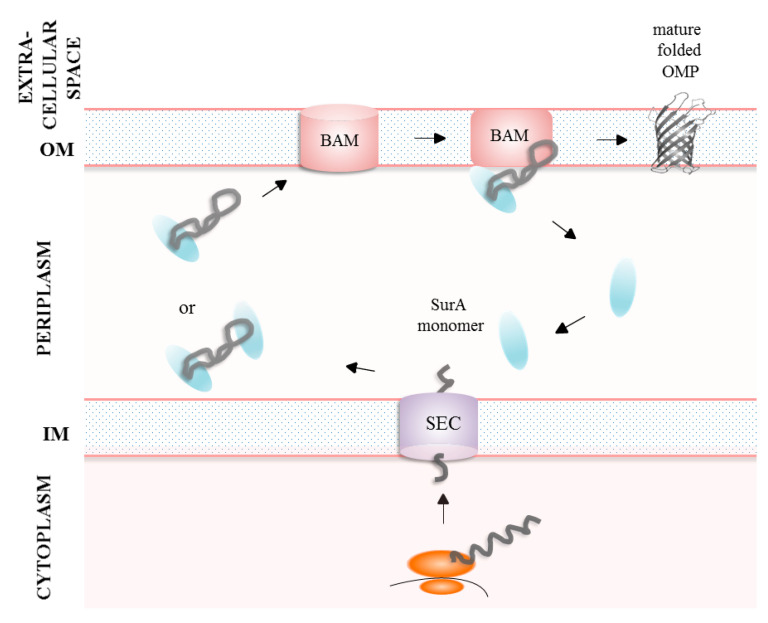
Model of SurA action. Under physiological conditions, SurA exists as a monomer. In the periplasm, OMPs are bound by one or more SurA molecules, depending on their size. OMPs are translocated to the BAM machinery in the OM where the interaction between SurA and BAM components facilitates efficient incorporation of the substrates into the OM. Once released from the complex, SurA is ready to bind another substrate molecule.

SurA does not form a classic cage around protected substrate [70]. Instead, the region between the core and P1 domains of the SurA monomer forms an OMP binding groove. The walls and base of the groove contain hydrophobic patches as well as positively charged regions. In turn, the surface near the top of the groove is negatively charged [69]. Upon binding to SurA, substrate undergoes conformational changes from more compacted to significantly expanded form [69,71,72]. This leads to exposition of other SurA binding segments allowing binding of more than one monomer of SurA to a client protein without steric clash [69]. Polypeptide expansion is thought also to prevent interchain contacts that may lead to substrate misfolding [71]. In case of small OMPs (up to 8 beta strands), the core domain itself is sufficient to shield substrate from aggregation [60]; however, for OmpC, the 40 kDa protein, the P2 domain assists in substrate holding and it strengthens SurA-OmpC interactions [73]. In the next step, the unfolded OMP shielded by SurA is translocated to the BAM machinery in the OM. The core and P2 domains are responsible for direct interactions between SurA and the BAM complex leading to conformational changes within the periplasmic regions of BAM components (BamA, BamB and BamE) and ensuring efficient folding of OMP into the OM [47]. After the process is completed, the released SurA monomers are free to encounter the next round of chaperoning. The nature of SurA-unfolded OMP interaction is transient which may be essential for efficient handover of OMPs to BAM for folding and insertion into the OM (Figure 4) [58]. Alongside the holdase function of SurA, the latest study revealed for the first time the ability of SurA to disassemble OMP aggregates. Moreover, SurA shows higher affinity for aggregated substrates than for unfolded ones, suggesting its important role against accumulation of aggregated proteins in periplasm under stressful conditions [71].

The SurA-like proteins are well conserved among proteobacteria, and they share a similar overall 3D structure [74]. In numerous bacterial species, they were shown to play a key role in OMP biogenesis. The *Yersinia surA* mutants, including *Y. enterocolica* [75], *Y. pestis* [76] and *Y. pseudotuberculosis* [77], show a reduced OM integrity and increased sensitivity to antibiotic treatment. In *Campylobacter jejuni*, the SurA-related chaperone PEB4 (also known as Cj0596 or CBF2) plays an important role in the OM biogenesis and integrity [78]. Mutation of *peb4* causes an alteration in the levels of eight OMPs, five of which are more abundant and three are less abundant in the mutant compared to wild type bacteria. Among them, components of flagella (FlaA and FlgE; increased level) and the adhesin CadF were identified [79]. In *Pseudomonas aeruginosa*, deletion of the *surA* gene is problematic and there are several reports suggesting that *surA* is essential [80,81,82]. However, recently it was shown that it is possible to inactivate the *surA* gene [83]; therefore, requirement for SurA may be strain–dependent in *P. aeruginosa*. The experiments using a conditional knockout of SurA demonstrated an altered abundance of 42 potential OM proteins and an increased OM permeability. The proteins of particularly lower content in the conditional mutant included several porins, TonB-dependent receptors and the siderophore receptors FpvA, FiuA and FecA, as well as T5SS proteins (e.g., Vd autotransporter PlpD) [80]. Moreover, it was demonstrated that SurA is essential for desiccation tolerance [83]. In *Bordetella pertussis*, the *surA* gene (*bp3330*) has not been inactivated thus far, indicating a crucial role of this protein in bacterial physiology [84,85]. On the other hand, the SurA-like protein does not seem to play a key role in the OMP biogenesis, LptD or antibiotic resistance in *Neisseria meningitidis* [86].

### 4.2. The Skp Protein

**Figure 5:**
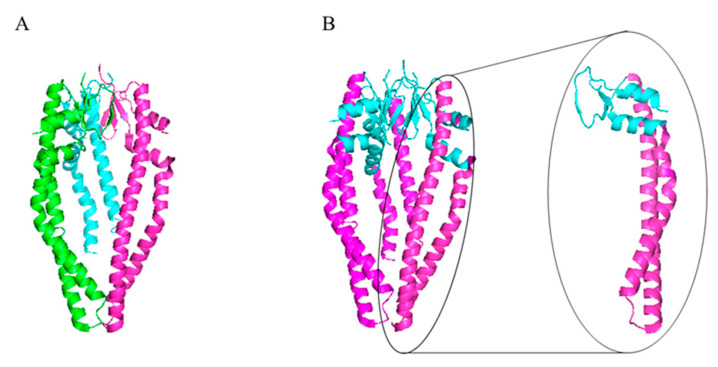
Crystal structure of the Skp trimer (PDB 1SG2). Side projections of the trimer: (**A**) each monomer represented in different color, (**B**) tentacles and association domain are colored magenta and cyan, respectively. The oval shows the monomer structure within the trimer—the monomeric Skp in solution is unstructured.

The Skp protein also known as OmpH is a small protein with a mass of 17 kDa, hence its name “Seventeen kilodalton protein” [54]. The Skp monomer is composed of two domains. The smaller one, the association domain, is formed by four beta strands and two short alpha helices and it enables oligomerization of Skp. The bigger one, the tentacle domain, folds almost completely into alpha helices, forming the long tentacle-shaped extensions characterized by conformational flexibility. The functional form of Skp is a trimer whose overall shape resembles a jellyfish (Figure 5) and bears similarity to the cytosolic chaperone prefoldin [87,88]. The “body” is made of β-barrels ensuring hydrophobic environment. Because the tips of tentacles are rich in positively charged residues, there are no interactions between tentacles from different monomers, and access to the central cavity for client protein is open from the bottom and on the sides.

**Figure 6:**
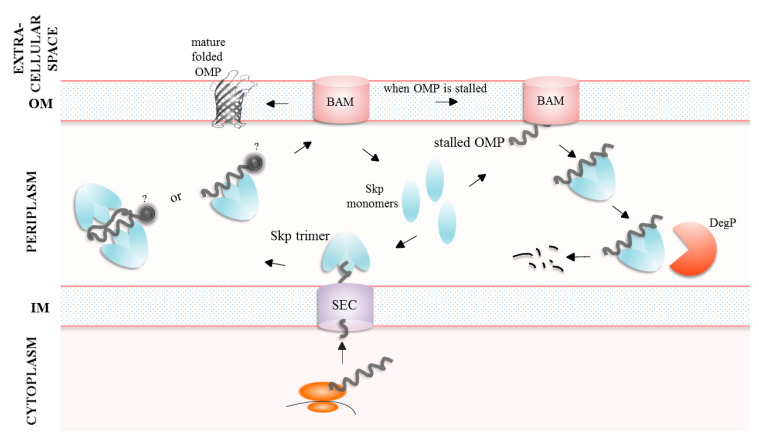
Model of Skp action. In the absence of substrate, Skp exists mainly in the monomeric state; in the presence of an unfolded polypeptide, the chaperone forms a trimer to encapsulate the substrate. Large or small substrates interact with Skp trimer with 2:1 or 1:1 stoichiometry, respectively. The periplasmic domain of an OMP is probably folded and exposed outside the trimer cavity (in the figure probability is represented with “?”). The Skp-unfolded OMP complex is transported to the BAM machinery. When OMP becomes stalled within the BAM machinery complex, Skp sequesters the defective OMP and directs it to degradation by the DegP protease; Skp is also degraded in this process.

Skp is involved in targeting proteins to the OM and it acts mainly by stabilization and possibly folding the client proteins (for details, see review [68]). Alternatively, Skp binds an unfolded/misfolded OMP, presents it to the DegP protease which degrades the whole complex. Hence, Skp may act as a sacrificial adaptor protein to degrade the aberrant OMPs [89]. The following model briefly summarizes the proposed mechanisms of SkpA action (Figure 6). In the periplasm, Skp exists in the monomer–trimer equilibrium, and at low molecular concentrations an inactive and intrinsically disordered monomeric form is favored. When an unstructured OMP polypeptide emerges from the SEC channel on the periplasmic side, the Skp monomers bind it and assemble around to form a trimer [90]. In an active trimer conformation, the hydrophobic patches localized on the tentacles interact with the unfolded β-barrel domain of a client protein, which becomes encapsulated within the inner cavity of Skp [90,91]. The periplasmic domains of the bound OMPs are most probably in a folded state and they protrude outside the Skp cavity. Small substrates interact with the Skp trimers with a 1:1 stoichiometry. In the case of large client proteins, two Skp trimers are necessary to provide sufficient protection from aggregation. The exact architecture of the 2:1 Skp:substrate complex remains unknown, but in simplified terms, the client protein is flanked on both sides by the Skp trimers [92]. The sequence of events during substrate release is unclear. It is not known whether the trimer should be destabilized before substrate release, or the trimer is destabilized only after the release of the client protein. In summary, the Skp chaperone activation mechanism is considered as disorder-to-order transition that is coupled with oligomerization, a combination so far not observed in other ATP-independent chaperones [90]. Recently, a novel model of Skp functioning has been proposed. According to it, the role of Skp is to directly facilitate degradation of the OMP substrates that are unable to properly insert and assembly into the OM. The Skp chaperone is necessary to sequester the defective OMPs from the BAM machinery and direct them for degradation by the DegP protease. Interestingly, Skp becomes degraded alongside with the bound OMP [89].

Skp displays a broad spectrum of substrates, mainly related to the OM biogenesis, including several virulence determinants (OmpA, OmpF, OmpC, LamB and OmpX) [93]. For example, in the uropathogenic *E. coli* strains, OmpX plays significant role in bacterial internalization and aggregation in mouse renal epithelial cells; however, is not necessary for adhesion to these cells. OmpX positively regulates transcription of *flhD* encoding a master regulator of flagellar expression. Deletion of *ompX* results in reduction in flagellar production and thereby lower motility [94].

It is generally believed that SurA plays a leading role in the biogenesis of the OM proteins and transit of autotransporters through the periplasm, while Skp plays a rather minor role. Most of these studies were performed on a model of *E. coli* and related bacteria. However, in case of other bacterial species, the importance of individual chaperones may be different, and it depends on the requirements of a specific client protein. The Skp homologs have been identified in various Gram-negative bacteria, underlying importance of the Skp function in the cell physiology. For example, in *N. meningitidis*, the absence of the Skp homolog, but not SurA homolog, resulted in lower levels of the major OMPs, the porins PorA and PorB [86]. In addition, considering the client proteins, the Skp homologs are expected to play important roles in bacterial virulence. An important virulence factor of *P. aeruginosa*, the extracellular lipase A, requires chaperoning by Skp prior to the secretion and in the absence of Skp the lipase secretion is nearly abolished [95].

## 5. Virulence-Related Processes Dependent on the SurA and/or Skp Functions

The proper delivery of OMPs to the OM is critical to the membrane integrity and its functionality. As mentioned previously, several important virulence-related processes such as adhesion and dissemination within the host, antibiotic resistance, evasion of the host immune systems, acquisition of nutrients and metal ions are at least partially dependent on the properly functioning OM in the Gram-negative bacterial pathogens. Therefore, these processes are in many cases disturbed in cells lacking SurA or/and Skp.

### 5.1. Adhession and Dissemination within the Host

The functions of SurA turned out to be very important for *E. coli* virulence, particularly uropathogenic *E. coli* strains (UPEC) [96]. UPEC invade superficial bladder epithelial cells via a mechanism dependent on the type 1 pili [97]. It was found that the UTI89 *surA* mutants were deficient in ability to adhere and invade the bladder epithelial cells, as well as to develop cystitis in the murine model. Initially, the defects in the infectivity of the UPEC *surA* knockouts were ascribed to disorders in the biogenesis of type-1 fimbriae, important virulence factors [98]. It was demonstrated that SurA is necessary to chaperone FimD, the integral OM fimbrial usher. Lower amount of the FimD usher in the OM results in an insufficient density of fimbriae on the cell surface [98,99]. Regarding the Skp chaperone, a direct link between the *skp* mutations and the *E. coli* virulence is still missing.

In *S. flexneri*, Skp and SurA play a role in intercellular spread. One of the spread determinants is IcsA, autotransporter associated with the cell surface. IcsA mediates actin polymerization on the bacterial surface in the host environment, providing the actin-based motility (ABM) through actin tail assembly. Ics-dependent ABM is a prerequisite for intercellular spread of *S. flexneri* throughout the colonic epithelium [100]. Mature IcsA consists of two domains: the alpha domain (effector domain), which is presented on the cell surface and enables actin polymerization, and the beta domain (translocation domain), which is required for protein incorporation into the OM. The level of the surface-presented IcsA is negatively regulated by the OM IcsP protease that hydrolyzes the junction between the alpha and beta domains. It was determined that deletion of the *skp* gene abolished plaque formation in the Henle cell monolayers due to a decreased presentation of IcsA on the cell surface, wherein the level of IcsA in the bacterial cell remained unchanged. In the absence of Skp, the IcsA beta domain was properly folded and autotransporter was incorporated into the OM at the same level as in the wild type (WT) background. This suggests that less effective presentation of IcsA on the surface of the *skp* mutant cells is due to improper folding of the alpha domain in the periplasm and its enhanced cleavage by the IcsP protease [101].

In *Y. enterocolica*, Yersinia adhesin A (YadA) and the adhesin Invasin (Inv) mediate binding to host cells via extracellular matrix proteins and/or ß1-integrins [102,103]. YadA and Inv belong to the autotransporter (AT) membrane protein family (T5SS); YadA is a type Vc AT and forms trimers while Inv is a monomeric, inverted type Ve AT. Both adhesins are affected by knockout of *surA* or *skp;* however, they are affected to a different extent [75].

In the case of *C. jejuni* NCTC11168 *peb4* mutant strain, adherence to INT407 cells was 1–2% that of the wild type strain. Proteomic analysis revealed that the levels of proteins involved in various adhesion and motility functions were lower in the *peb4* mutant than in the wild type strain [104]. Opposing observations were presented in the subsequent work [105] where it was shown that the *peb4* mutation does not affect adherence to the INT407 cells. Therefore, the effect of the *peb4* mutation on adherence may be strain-dependent. It was determined that in the absence of the SurA-like protein, the level of the CadF protein is decreased [79]. CadF is known to promote binding to fibronectin on the host cells and is required for maximal adherence and invasion of INT407 cells and colonization of the chicken cecum [106]. Interestingly, deletion of *peb4* was shown to increase motility of *C. jejuni* [105].

### 5.2. Antibiotic Resistance

The OM of Gram-negative bacteria provides a physical barrier that efficiently limits the entry of antibiotics into the cell [8]. As the SurA and Skp proteins are involved in the OM biogenesis and their lack affects the OM composition and integrity, the *surA* and *skp* mutations are frequently associated with altered antibiotic resistance. The *Y. enterocolica* Δ*surA* mutant strain shows an increased sensitivity to four antibiotics tested (rifampicin, erythromycin, vancomycin and bacitracin) as compared to the wild type strain, while the Δ*skp* mutant was more sensitive to erythromycin compared to the wild type strain and vancomycin had subinhibitory effect on it [75]. Two other *Yersinia* species, *Y. pestis* and *Y. pseudotuberculosis*, also show increased susceptibility to antibiotic treatment [76,77]. *C. jejuni peb4* mutant was more susceptible to ampicillin and vancomycin treatment [79].

### 5.3. Evasion of the Host Immune Systems

Survival within the host organism and successful establishment of infection frequently is associated with the ability of a pathogen to evade the host immune defenses. The simplest way is to avoid detection. For example, UPEC form intracellular bacterial communities inside the epithelial cells and this way avoid recognition by the host immune systems [107,108]. A lack of SurA affects the growth and survival of UPEC in the host cells and the mutant bacteria fails to establish the intracellular bacterial communities [98]. It was demonstrated that at least one of the SurA substrates, OmpA, is involved in the intracellular bacterial community formation and intracellular survival of UPEC [109].

Pathogens are also able to manipulate the host immune responses. It was determined that the cystitis-derived UPEC strains (UTI89 and NU14) are capable to suppress the responses of the bladder cell lines to exogenous LPS. Interestingly, mutations in LPS biosynthetic genes or in the surA gene result in the significantly higher cytokine induction from bladder epithelial cells [110]. As mentioned previously, a lack of SurA is associated with abnormalities in the LPS protective barrier [62]. Therefore, it was hypothesized that an inappropriate LPS organization in the *surA* mutant cells may lead to the increased availability of lipid A for recognition by host cell surface receptors [110]. However, subsequent reports demonstrated that UPEC suppression of IL-6 secretion is not mediated by LPS and suggested a more complex mechanism [111]. For example, an additional effector molecule may be involved. It was shown that the periplasmic protein YbcL expressed by UPEC is able to suppress transepithelial neutrophil migration in the murine model of cystitis [112]. Whether YbcL functioning is dependent on SurA remains to be explored. Nevertheless, it cannot be excluded that a SurA-dependent secreted or surface-exposed effector (yet unknown) is responsible for cytokine suppression by wild type UPEC.

The serum complement system is an important first line innate host defense against invading microorganisms [113]. A number of the literature reports link the lack of SurA and Skp functions with increased sensitivity to serum killing. In *P. aeruginosa*, depletion of SurA (but not Skp) results in a lower survival in active serum [80]. The *Y. enterocolica surA* or *skp* mutants are more sensitive against human serum than the wild type bacteria [75]. In *Yersinia*, YadA is a major factor that determines serum resistance by affecting negative regulators of the component cascade, including factor H, C3b, iC3b, and vitronectin [114,115,116,117,118]. Although SurA and Skp were shown to interact with YadA, the OM levels of this protein are unchanged in the mutant *Y. enterocolica surA* or *skp* strains [75]. Therefore, the exact reason of lower serum resistance of the mutant strains remains to be discovered.

Not always does a lack of a chaperone lead to an increased sensitivity to host immune systems. In *Salmonella enterica* serovar Typhimurium, it was shown that the deletion of Skp leads to a hyposensitivity against antimicrobial peptides (AMPs) [119]. The mechanism of the *skp*-dependent AMP resistance is not understood to date.

### 5.4. Acquisition of Nutrients and Metal Ions

Uptake of sufficient amounts of nutrients is crucial for bacteria to grow and multiply. However, in the host’s organism, access to these compounds is limited. In order to acquire nutrients, bacteria secrete dedicated proteins and other compounds such as siderophores and utilize the OM uptake and transport systems. Proteases, lipases, phospholipases and other hydrolases degrade the host’s macromolecules and thus provide nutritional particles suitable for uptake [120]. These enzymes are in many cases secreted by the T2SS or T5SS, and therefore may require assistance of the periplasmic chaperones, including SurA and Skp. A well-described example is the autotransporter haemoglobin protease (Hbp) which a key virulence factor of pathogenic *E. coli* strain EB1 causing a severe inflammation of the peritoneal cavity [121]. Hbp degrades human haemoglobin and binds the released heme, making it accessible for bacteria [122]. Hbp is a Va type of T5SS which transiently interacts with SurA upon its periplasmic transit [123]. SurA and/or Skp can affect acquisition of iron also at the level of the OM receptors. In the *surA* or *skp* mutants, the aberrant levels of the OM iron uptake components are observed. For example, the OM of the *P. aeruginosa surA* mutant is deficient in receptors for siderophores, FpvA, FecA, and Fiu [80]. Likewise, *C. jejuni peb4* mutant contains lower levels of an iron uptake protein, FepA, and haemin uptake system outer membrane receptor, CirA [104], *Y. enterocolica surA* or *skp* mutants show decreased level of an iron uptake receptor, FyuA [75].

### 5.5. Secretion of Toxins

The serine protease EspP produced by the enterohaemorrhagic *E. coli* (EHEC) is an important virulence factor which degrades human coagulation factor V [124], stimulates host actin remodeling and shiga toxin micropinocytosis [125], and facilitates intestinal colonization [126]. This protein is a type Vc of T5SS whose biogenesis is partially dependent on the periplasmic chaperones. Using various experimental approaches (photocrosslinking or surface plasmid resonance), it was demonstrated that periplasmic chaperones SurA and Skp bind not only the “translocator” β-barrel but also the “passenger” domain of EspP [127,128].

The plasmid-encoded toxin (Pet), an AT produced by enteroaggregative *E. coli* (EAEC), binds to Skp and another periplasmic chaperone, VirK. Skp interacts specifically with the β-barrel translocation domain of Pet and may facilitate incorporation of Pet into the OM. Although in the absence of Skp, Pet β-barrel domain is still inserted the into the OM and the Pet “effector” is secreted into the medium, both events occur with lower efficiency than in the isogenic wild type strain [129].

### 5.6. Biofilm Formation

Formation of biofilm is one of the strategies to survive unfavorable environmental conditions, including these in the host organism. Moreover, it protects bacterial cells against the host’s immune system and antimicrobial substances (reviewed in [130]). The *S. enterica* serovar Typhi *surA* mutant exhibited significantly reduced ability to form biofilms compared with the wild type bacteria. It was demonstrated that the mutation affects flagella expression via the RcsCDB pathway and thereby affects biofilm formation [131]. Likewise, impaired biofilm formation was reported in *C. jejuni peb4* [104], uropathogenic *E. coli* UTI89 *surA* [132].

As a consequence of one or more of the aforementioned defects, the *surA* and *skp* mutants are frequently attenuated in the animal models of infection. Some well-documented examples are listed below. The *surA* UPEC knockouts are not able to develop cystitis in the murine model of infection [98]. In *Y. enterocolitica*, SurA and Skp both play role in systemic and oral infection of mouse; however, it is important to note that the effects of the *surA* deletion were much more pronounced than those of *skp.* In the systemic mouse infection model, the *Y. enterocolica* Δ*surA* and *Δskp* mutants do not replicate at the wild type levels. Furthermore, they are cleared more efficiently by the host. In the oral infection model, the Δ*surA* mutants can reach the gut but are unable to disseminate and are cleared. The Δ*skp* mutants are able to enter the Peyer’s patches from the gut but cannot further disseminate to the mesenteric lymph nodes. No mutant bacteria were detected in the spleen five days after oral infection [75].

In *S. enterica* serovar Typhimurium, deletion of *skp* resulted in significant attenuation in a murine typhoid model. However, the link between *Salmonella* virulence and Skp protein is not clear. The in vitro data did not show growth differences between the mutant and wild type strain in under thermal or oxidative stresses, as well as in the presence of polymyxin B. Functioning of Type III secretion system was also not distorted [133].

In *C. jejuni*, deletion of the *peb4* gene results in attenuation of virulence in a mouse model [104,105]. A decreased virulence due to a lack of SurA was also observed in *S. flexneri* [134] and *S. enterica* [135,136,137]. The *surA* deletion mutant of *S. flexneri* is incapable of plaque formation in Henle cell monolayers. It is most probably associated with the proper folding and insertion of IcsA into the OM [134]. Deletion of *surA* decreases the ability of *S. enterica surA* to invade Caco-2 and RAW264.7 cells in vitro, and results in lower capacity to colonize BALB/c mice [137].

## 6. SurA-like Proteins as the Extracellular Virulence Factors

Although SurA and Skp-like proteins localize generally to the periplasm, in case of some bacteria, including pathogenic species, they are also found in the extracellular space. Hence, aside from important intracellular functions, the SurA-like proteins can play direct important roles in bacterial virulence as secreted virulence factors. A well-documented example is the *H. pylori* SurA-like HP_0175 protein (using *H. pylori* 26,695 strain nomenclature), known also as *H. pylori* cell binding factor 2 (HpCBF2). This protein was shown to be a proapoptotic factor [138], as a significant reduction in the induction of apoptosis by the *H*. *pylori* mutant lacking HP_0175 was observed. Furthermore, it was demonstrated that HP_0175-induced cell death depends on apoptosis signal-regulating kinase 1 (ASK1), MAPKs, and caspases. HP_0175 was able to interact directly with Toll Like Receptor 4 (TLR4), which is expected to be a first step in the HP_0175-dependent apoptosis pathway. As a consequence, ASK1 becomes activated, leading to the sequential activation of p38 MAPK and caspase 8. The apoptotic signals are further amplified through the mitochondrial pathway involving cleavage of the Bid protein and subsequent translocation of truncated Bid to the mitochondria, followed by the loss of mitochondrial membrane potential, cytochrome c release, and sequential activation of caspases 9 and 3 [138]. Binding of HP_0175 to TLR4 is linked to another important process observed in the *H. pylori*-associated diseases. Specifically, it triggers the translocation of TLR4 to lipid rafts, where TLR4 becomes phosphorylated by kinase Lyn. Phosphorylated TLR4 interacts with epidermal growth factor receptor (EGFR) which leads to activation of this receptor and subsequent stimulation of EGFR-dependent vascular endothelial growth factor (VEGF) production [139]. The observations presented above point to a possible relationship of HP_0175 with pathophysiology of ulcerogenesis and/or carcinogenesis. Therefore, HP_0175 is considered a potential target for novel antimicrobial drug design.

Other surface exposed or secreted SurA-like PPIases have been reported: *Leptospira interrogans* LIC12922 [140], *B. pertussis*, the SurA-like protein (BP3330) [141], *Vibrio parahaemolyticus* SurA and Skp homologs [142]. However, in these cases, involvement of the SurA-like or Skp-like proteins in virulence is unknown.

## 7. SurA and Skp-like Proteins as Therapeutic Targets

The components of the periplasmic PQCS are in many cases key factors determining virulence of numerous important Gram-negative pathogens. For this reason, attempts are being made to construct molecules that disrupt the functioning of this system as a strategy to combat pathogenic microorganisms (reviewed in [14,75,143,144,145]).

There is increasing evidence pointing at the virulence-associated functions of the SurA and/or Skp-like proteins in various pathogenic bacteria; therefore, these proteins are of increasing interest as potential therapeutic targets to replace or support antibiotic treatment. As described in previous sections, a lack of SurA is generally associated with severe phenotypes and it causes reduced virulence of most examined bacterial pathogens. Moreover, the SurA functions seem to be associated with antibiotic resistance [80]. Consequently, it was SurA that became the first target among the periplasmic chaperones to search for inhibitory molecules. The *E. coli* SurA protein was used as model to screen over 10,000 compounds in silico [146]. The authors were able to initially select twelve commercially available compounds which were predicted to bind to the putative client binding site in the SurA molecule. Experimental verification of binding led to selection of one compound, Fmoc-β-(2-quinolyl)-_D_-alanine, which was then used as a lead. Subsequent experiments allowed for the selection of two additional compounds: Fmoc-_L_-tryptophan and Fmoc-_L_-phenylalanine. Although these compounds show rather low affinity to SurA and are not potent inhibitors, they should provide a good starting point for further modifications.

It is also worth mentioning that the SurA-like proteins are strong antigens and research is underway to use them as vaccine components. For example, immunization with *Brucella* SurA protects mice from challenge with *B. abortus* [147]. Alternatively, *surA* knockout strains may become effective vaccines, as demonstrated for *S. enterica* serovar Typhimurium *surA* mutants [135].

In addition, HP_0175 (HpCBF2), which is a highly reactive antigen found in sera of the *H. pylori* infected patients [148], was proposed to serve as a potential marker for gastric disease-associated *H. pylori* strains [149].

## 8. Conclusions

Correct biogenesis of the OM and efficient secretion of active virulence factors are necessary for successful infection and development of disease symptoms in the host organism. Consequently, disturbances in these processes often lead to the attenuation of bacterial virulence. For this reason, much attention is paid to proteins that allow both processes to take place. These include the SurA-like and Skp chaperones which play important roles at the stage of transporting secretory proteins across the periplasm but also may be involved in the incorporation of integral membrane proteins to the OM. This makes them promising therapeutic targets. The SurA-like proteins seem to be particularly attractive targets, considering importance of their functions in the bacterial cell physiology but also their extracellular functions as direct virulence factors. Although there are no efficient inhibitors against chaperone activity of SurA, research to date has provided preliminary data that could become a starting point for the development of specific and potent inhibitory molecules.

## Data Availability

Not applicable.
